# Optically Active Nanomaterials for Bioimaging and Targeted Therapy

**DOI:** 10.3389/fbioe.2019.00320

**Published:** 2019-11-15

**Authors:** Yu Yang, Li Wang, Bin Wan, Yuxin Gu, Xinxin Li

**Affiliations:** ^1^State Key Laboratory of Environmental Chemistry and Ecotoxicology, Research Center for Eco-Environmental Sciences, Chinese Academy of Sciences, Beijing, China; ^2^College of Resources and Environment, University of Chinese Academy of Sciences, Beijing, China; ^3^Rural Energy and Environment Agency, Ministry of Agriculture, Beijing, China

**Keywords:** optical, nanomaterials, bioimaging, tissues, therapy

## Abstract

Non-invasive tracking for monitoring the selective delivery and transplantation of biotargeted agents *in vivo* has been employed as one of the most effective tools in the field of nanomedicine. Different nanoprobes have been developed and applied to bioimaging tissues and the treatment of diseases ranging from inflammatory and cardiovascular diseases to cancer. Herein, we will review the recent advances in the development of optics-responsive nanomaterials, including organic and inorganic nanoparticles, for multimodal bioimaging and targeted therapy. The main focus is placed on nanoprobe fabrication, mechanistic illustrations, and diagnostic, or therapeutical applications. These nanomedicine strategies have promoted a better understanding of the biological events underlying diverse disease etiologies, thereby facilitating diagnosis, illness evaluation, therapeutic effect, and drug discovery.

## Introduction

Molecular imaging reflects biological information on temporal and spatial scales, unveiling the dynamics of disease (Smith and Gambhir, 2017). It is a crucial diagnostic tool for monitoring *in vivo* response and assessing outcomes in targeted therapies (Fan et al., 2017). Also, integrating imaging, and therapy into theranostic systems is an efficient strategy for real-time tracking of the pharmacokinetics and biodistribution of a drug. Current imaging modalities include X-ray, magnetic resonance, optics (e.g., fluorescence, luminescence, Raman, photoacoustics), radionuclides, and mass spectrometry (Kunjachan et al., 2015). Among them, optical imaging is a common modality in preclinical research on theranostic agents.

Nanomaterials have been widely developed as therapeutic and diagnostic agents (Lim et al., 2015; Chen et al., 2016a). Research efforts have changed from developing new materials *in vitro* to exploring functional materials *in vivo*, thereby increasing the potential for clinical translation (Lamch et al., 2018). Perhaps the unique advantage of nanoparticles (NPs) for optical imaging is that their physical and optical properties are easily tunable through structural modulation. Also, some NP compositions possess inherent imaging and therapeutic properties, while others are rendered multifunctional through the manipulation of multiple structural elements. Engineering multifunctional theranostic NPs presents several challenges, including imaging quality, loading capacity, the toxicity of intrinsic ingredients, storage, and *in vivo* stability, the complexity of synthesis, batch repeatability, production costs, and regulatory hurdles (Farokhzad and Langer, 2006; Lee et al., 2012). Common nanomaterials, including inorganic and organic NPs, have demonstrated a potential for diagnosis and therapy (Brigger et al., 2002). Variations in size, shape, and surface modifications can adjust their biocompatibility and specificity with target tissues (Wang and Thanou, 2010). Depending on their structural composition, NPs can provide an optical signal or function as nanocarriers for optically active agents. Current interests mainly involve non-invasive imaging of deep tissues and targeting drug therapy.

In this paper, we discuss recent progress in optical-sensitive NPs, their bioimaging involving fluorescence, luminescence, surface-enhanced Raman scattering (SERS), and photoacoustic (PA) signals, and their therapeutic applications in photodynamic therapy (PDT), photothermal therapy (PTT), and drug delivery. Moreover, common design considerations for advanced nanomedicines and the challenges of their application are discussed from diagnostic and therapeutic perspectives.

## Optically Active Nanomaterials

### Inorganic Nanomaterials

Due to their unique characteristics, i.e., surface plasmon resonance (SPR), gold NPs (GNPs) are usually chosen to enhance optical imaging based on their absorption, fluorescence, Raman scattering, etc. (Wu et al., 2019). Generally, GNPs are synthesized by HAuCl_4_ reduction, known as the Brust et al. (1994) or Turkevich method Turkevich et al. (1951). GNPs are stabilized by a wide variety of ligands that affect their sizes and properties (Treguer-Delapierre et al., 2008; Boisselier et al., 2010). Their diameters range from 1 nm to more than 120 nm. Also, diverse shapes can be prepared, such as core–shell nanostructures (Kharlamov et al., 2015), nanorods (de la Zerda et al., 2015), or nanocages (Chen et al., 2005a) whose aspect ratios modulate their optical properties. The excellent stability of GNPs covalently bonded with thiolated ligands permits chemical modifications directly on their surfaces (Boisselier et al., 2008). The ligands for stabilizing GNPs can be specifically selected for drug encapsulation and release or targeted to tissues such as tumors (Guo et al., 2017; Her et al., 2017; Spyratou et al., 2017). However, the safety of GNPs in clinical application remains controversial, with more information required on their long-term toxicity *in vivo*.

Carbon-based nanomaterials (CBN) as members of the carbon family including fullerenes, carbon nanotubes (CNTs), graphene (G), graphene oxide (GO), nanodiamonds (NDDs), and carbon dots (CDs) (Bartelmess et al., 2015; Patel et al., 2019). Modifying their surfaces with functional groups (e.g., carboxylic acid, hydroxyl, or epoxy) provides the opportunity to optimize their properties. The extraordinary optical features of CBN (e.g., inherent fluorescence, high photostability, and tunable narrow emission spectra) increase their potential for imaging and diagnosis of cells or tissues (Sadegh and Shahryari-ghoshekandi, 2015; Goodarzi et al., 2017; Namdari et al., 2017; Bullock and Bussy, 2019; Tinwala and Wairkar, 2019). Fullerenes usually act as photosensitizers (PSs) to generate singlet oxygen (^1^O_2_) and hence are applied for blood sterilization and cancer PDT (Lu et al., 2019). The inherent spectroscopic features of CNTs (e.g., Raman scattering and photoluminescence) provide a valuable means of tracking *in vivo* therapeutic status, pharmacodynamic behavior, and drug delivery efficiency and imaging and detecting diseases (Tasis et al., 2006; Liu et al., 2011). Graphene and GO-based nanocarriers have attracted significant attention for imaging and anticancer therapy because of their large drug loading and effective delivery capacity. Also, ~2,600 m^2^/g is more than double the surface area of most nanomaterials (Mao et al., 2013; Reina et al., 2017). Recently, carbon dots (CDs, size <10 nm) have been extensively studied to gain a high fluorescence quantum yield through facile synthesis methods (Liu et al., 2015a). NDDs are nanocrystals that consist of tetrahedrally bonded carbon atoms in the form of a three-dimensional (3D) cubic lattice. The optical properties of NDDs allow their use as photoluminescent probes (λ_em_ = 550–800 nm) due to nitrogen-vacancy defect centers (Chang et al., 2008). When functionalized, their biocompatibility is known to be superior to CNTs and carbon black (Mochalin et al., 2013). However, the toxicity of CBN is presently the key problem for their clinical use. Also, the toxicology and pharmacokinetics of CBN mainly rely on several factors, e.g., physicochemical and structural properties, exposure dose and time, cell type, mechanism, residual catalyst, and synthesis method. It is necessary to systematically evaluate CBN *in vivo* safety using more relevant animal models.

Porous silicon nanoparticles (pSiNPs) have gained intense attention in the biomedical field due to their low toxicity and potential for use in minimally invasive and focal therapies that avoid conventional side effects (Vivero-Escoto et al., 2012). pSiNPs can be degraded completely to produce non-toxic orthosilicic acid (Tzur-Balter et al., 2013), a bioavailable form of Si, and then excreted efficiently through renal clearance (Park et al., 2009). Also, their high active surface areas (up to 1,000 m^2^/g) (Loni et al., 2015) allow Si modifications by other molecules through various surface chemical reactions (e.g., hydrosilylation, silanisation, and hydrocarbonization), which facilitate their targeted and controlled drug release into cancer cells (Wu et al., 2011a; Makila et al., 2012; Wang et al., 2014). Actually, the porous nanostructures of pSiNPs allow them to load drugs, probes, enzymes, proteins, antibodies, siRNA, or other species (Castillo and Vallet-Regi, 2019). Numerous cell-specific epitopes and biomarkers afford selective binding to certain antibodies, peptides, or other molecules and thus provide the opportunity for targeted drug delivery via vectorized pSiNPs (Li et al., 2018a). Moreover, the optical features of silicon nanostructures, i.e., intrinsic photoluminescence, afford an alternative for bioimaging, accompanied with better biocompatibility and biodegradability and lower toxicity compared with semiconductor quantum dots (Warner et al., 2005; Erogbogbo et al., 2008; Bimbo et al., 2010; Gu et al., 2010). Finally, easy handling and cost-efficient preparation through facile electrochemical anodization of crystalline silicon increase the interest in utilizing pSiNPs (Sailor, 2012). Nevertheless, the design and synthesis of smart multifunctional pSiNP nanocarriers, *in vivo* performance evaluation using animal models, and subsequent translational studies still need to be further explored.

Lanthanide (Ln)-doped upconversion nanoparticles (UCNPs) can convert two or more low-energy near-infrared (NIR) photons into high-energy emissions through a non-linear anti-Stokes process (Auzel, 2004). Due to their special upconversion luminescence feature and stable Ln-based inorganic framework, Ln-UCNPs have been developed as promising alternatives to conventional labels (e.g., organic dyes and quantum dots) with several attractive advantages, such as being highly resistant to photobleaching and photoblinking, having negligible background autofluorescence, having deeper tissue-penetration ability, and causing minimal photodamage (Zhou et al., 2012). Also, considering the high feasibility and maneuverability of their structural design, many functional components with other imaging capabilities are introduced into these nanoparticles for diagnostic and therapeutic applications (Liu et al., 2014a, 2015b). Likewise, UCNPs with a high surface area, characteristic structures, and versatile surface functionalization can act as nanocarriers for drug delivery (Wu et al., 2015; Yang et al., 2015a). Significantly, UCNPs with a distinctive upconverted emission can double as light-transducers to activate PSs for deep-tissue PDT or as phototriggered devices for precise control of drug-payload release induced by NIR light (Wang et al., 2013a; Min et al., 2014; Idris et al., 2015). However, high upconversion efficiency is realized only upon 980- or 808-nm excitation, which has seriously hindered their practical usage in the biomedical field. Also, surface modification and bioconjugation of long-term stable and biocompatible Ln-UCNPs remain a huge challenge. Therefore, more strategies for surface modification and corresponding toxicology studies of these NPs are still needed for future pre-clinical and clinical applications.

Quantum dots (QDs) are semiconductor nanocrystals (typically with diameters <10 nm), which display dimension-dominant optical features (e.g., absorption and luminescence) (Xu et al., 2018a). Particularly, QDs of various dimensions or ingredients are excited by a single light source to separately emit diverse colors over a wide range with negligible spectral overlap, rendering them desirable for multiple imaging (Yong, 2012). Avoiding the photobleaching of organic dyes, QDs are more beneficial to imaging *in vivo* for a long time (Geszke-Moritz and Moritz, 2013). Significantly, QDs can be tuned to emit over a broad wavelength range (e.g., 450–1,500 nm) by modulating their dimensions, configurations, and components (Pichaandi and van Veggel, 2014; Zhao et al., 2018). QDs can also be conjugated with a recognition molecule, such as antibodies, DNA, proteins, etc. (Lu and Li, 2011). Flexibility in the surface chemistry and emission peak of QDs makes them useable as optical probes or nanocarriers for bioimaging, drug discovery, diagnostics, and therapy (Probst et al., 2013; Bilan et al., 2016; Yao et al., 2018). The main QDs involved in biomedicine contain Cd-based QDs (e.g., CdSe, CdTe, CdS) or Cd-free QDs (e.g., InP, CuInS_2_, AgInS_2_, Ag_2_S, WS_2_, ZnO, silicon, GQDs, CuS) (Xu et al., 2016; Mo et al., 2017). However, the potential hazards of QDs have become a critical issue that must be further addressed prior to clinical use.

### Organic Nanomaterials

Optical nanoagents (e.g., QDs, UCNPs, metal nanoclusters, carbon, and silica-based nanomaterials) have been widely employed for molecular imaging. However, inorganic NPs usually suffer from critical safety concerns, including heavy metal poisoning, and not being susceptible to fast clearance from the body. To facilitate clinical translation, organic systems are generally preferred.

Fluorogens with aggregation-induced emission (AIEgens), the typical organic nanomaterials, exhibit very weak emission in the molecular state but high fluorescence in the aggregated state (Luo et al., 2001). They are usually arranged in a rotor-like conformation. In the free state, intermolecular collisions cause the consumption of energy in the form of non-radiative transition, while in the aggregated state, the restriction of intramolecular motion (RIM) ensures the release of exciton energy via a radiative pathway, intensifying the emission (Mei et al., 2015). Through intelligent modulation, AIEgens can emit a broad wavelength in the ultra-violet (UV) to NIR region, accompanied with certain attractive qualities. For example, they enhance the chance of intersystem crossing, thereby increasing the production of free radicals for PDT (Hong et al., 2009; Xu et al., 2015) and strengthen the conversion of non-radiative relaxation to generate thermal energy for PA imaging and PTT (Mei et al., 2014; Geng et al., 2015). Also, some biomolecules (e.g., target or chelation agents) can be integrated into AIEgens without affecting their AIE properties (Hong et al., 2011; Hu et al., 2014), which endows them with multiple performances for cancer therapy (Yuan et al., 2015). AIEgens have been successfully combined with PSs, drugs, and several imaging methods to realize efficient theranostic platforms (Chang et al., 2012; Xue et al., 2014). Most AIE systems with multiple functions (or modalities) are composed of several components through an all-in-one approach, suffering from defects in their complex molecular structure and multistep synthesis.

Owing to poor photostability of traditional NIR organic dyes, organic semiconducting agents, including semiconducting polymer nanoparticles (SPNs), and semiconducting molecule nanoparticles (SMNs), have emerged as excellent candidates (Lyu et al., 2016; Pu et al., 2016; Zhu et al., 2016). These agents possess higher absorption coefficients, more tunable optical properties, and controllable dimensions compared with inorganic agents (Hong et al., 2012a; Pu et al., 2014a). The wavelength range 650–1,000 nm of NIR fluorescence imaging is known as the first NIR window (NIR-I), which can achieve a tissue penetration depth of up to ≈1 cm (Shanmugam et al., 2014). However, when NIR-I light passes through or interacts with the tissue, it still suffers from light scattering, tissue absorption, and autofluorescence (Ntziachristos, 2010), leading to a relatively poor signal-to-noise ratio (SNR), and thus is less ideal for deep-tissue imaging. To enhance the imaging depth, the second near-infrared window (NIR-II, 1,000–1,700 nm), a more ideal region with deeper tissue-penetration depth and fewer light–tissue interactions, has been explored recently (Miao and Pu, 2018; Cai et al., 2019). To date, organic semiconducting agents have been utilized for deep-tissue imaging, including NIR-II fluorescence, self-luminescence, and PA imaging, in biomedical fields such as cell (Wu et al., 2010; Pu et al., 2014b), tumor (Wu et al., 2011b), acute edema (Pu et al., 2014b), and cardiovascular imaging (Hong et al., 2014) and in real-time evaluation of drug-toxicity (Shuhendler et al., 2014). However, they also face the major issues of high accumulation in the liver and slow removal from the body due to their large dimensions. Therefore, new designs are needed to increase their biodegradability or reduce their sizes so that they are smaller than the renal filtration threshold (~5 nm) for quick clearance via urine excretion.

Polymer nanoparticles (PNPs) have been widely developed for cancer diagnosis and therapy due to their high extinction coefficients, extraordinary fluorescence intensity, good photostability, biocompatibility and biodegradability, and simple encapsulation of anticancer drugs and/or imaging probes (Pecher and Mecking, 2010; Yang et al., 2012; Feng et al., 2017). Although PNPs can accumulate in the tumor region via the enhanced permeability and retention (EPR) effect (Luk and Zhang, 2014; Tang et al., 2016), cancer-targeting moieties (e.g., proteins, peptides, and aptamers) must be modified onto the NP surface by chemical or physical interaction, resulting in functionalized PNPs with enhanced selectivity and sensitivity for cancer diagnosis and therapy (Liu et al., 2015c; Li et al., 2016; Li and Yang, 2017; Lin et al., 2017). Recently, functionalized PNPs have been designed according to specific cellular processes and disease environments and applied to selectively deliver drugs to the target regions (Vaidya et al., 2011; Krasia-Christoforou and Georgiou, 2013; Herranz-Blanco et al., 2016; Kumar et al., 2018; Wang et al., 2019). Further *in vivo* work will be required to comprehensively investigate the safety and efficacy of these novel theranostic platforms before clinical application.

## Optically Active Nanomedicine for Bioimaging

### Fluorescence Imaging

Fluorescence imaging, as a means to visualize specific organelles within cells or animals, has become a powerful tool for biological research and even for the emerging field of fluorescence-guided surgery. Additionally, fluorescence imaging can capture specific molecular information on the tumor microenvironment (Wolfbeis, 2015).

**Figure 1:**
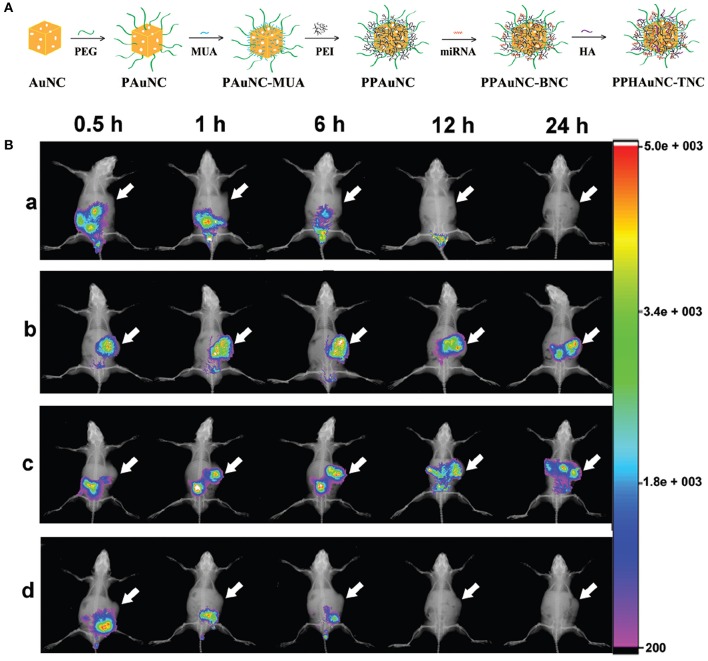
**(A)** Formation of PPHAuNC-TNC. **(B)** *in vivo* biodistribution of differently-sized PPHAuNCs-TNCs. After intravenous injection with (a) naked Cy5.5-miRNAs (control group), (b) PPHAuNCs-30-TNCs, (c) PPHAuNCs-50-TNCs, and (d) PPHAuNCs-70-TNCs.

Among the commonly utilized non-viral vectors, gold nanocages (AuNCs) were investigated for microRNA (miRNA) delivery due to their biological inertness and unique physicochemical features (Skrabalak et al., 2008). Their strong and easily adjustable absorption and scattering in the NIR region demonstrate their feasibility in theranostic uses (Xia et al., 2011). The sizes, shapes or surface chemistry of NPs can mediate their cellular uptakes, macrophage clearance, and biodistribution (Huang and El-Sayed, 2010). NPs with a size of 10–100 nm are more appropriate for cancer diagnosis and therapy since they can escape from renal elimination and accumulate in tumors. Besides size, surface chemistry is another crucial factor for the cellular uptake and biodistribution of NPs *in vivo* (Chen et al., 2005b). Bao et al. (2017) have successfully fabricated a series of differently-sized miRNA delivery systems, miR-26a-loaded, hyaluronic acid (HA)-modified, polyetherimide-conjugated PEGylated AuNC ternary nanocomplexes (PPHAuNCs-TNCs), and monitored them through fluorescence and PA imaging. First, they prepared PEGylated AuNCs (PAuNCs) by employing HS-PEG-OMe (MW 5000) to replace the PVP layer through an Au-S bond to extend the circulation period of the NPs in the blood and enhance their stability. Secondly, polyethyleneimine (PEI)-conjugated PAuNCs (PPAuNCs) were obtained by conjugation of PEI onto the surface of the PAuNCs to condense miRNAs, utilizing 11-mercaptoundecanoic acid (MUA) as a linker. Thirdly, miRNAs were encapsulated with differently-sized PPAuNCs via electrostatic interaction to obtain miRNA-loaded, PEI-conjugated PEGylated AuNC binary nanocomplexes (PPAuNCs-BNCs). Finally, to neutralize their slightly positive charge and enhance their stability and biocompatibility, a small amount of HA was added to PPAuNCs-BNCs to obtain the final product, PPHAuNC-TNC (Figure 1A).

miRNAs were labeled with Cy5.5, a NIR fluorescent dye, so as to visualize the effect of NP size on biodistribution *in vivo*. Figure 1B shows real-time images of differently-sized PPHAuNCs-TNCs in BEL-7402 tumor-bearing nude mice. Notably, the fast tumor accumulation of PPHAuNCs-30-TNCs was clearly observed at 0.5 h and approached saturation at 6 h post-injection. For the PPHAuNCs-30-TNC group, sustainable maintenance of the relatively high fluorescence density was found at the tumor site at 24 h, which was similar to the PPHAuNCs-50-TNC group. However, slower and less tumor accumulation was observed for the PPHAuNCs-50-TNC group than for the PPHAuNCs-30-TNC group. In addition, no obvious fluorescence signals were detected at the tumor site 24 h after injection in the PPHAuNCs-70-TNC and naked Cy5.5-labeled miRNA groups. This was likely due to the accumulation at the tumor site being too weak to be examined or its rapid clearance from the body. The different biodistribution profiles of the three NPs clearly indicated that NP accumulation in the tumor could be ascribed to not only the EPR effect but also diffusion, which largely relied upon the morphology, dimensions, and surface charges of the NPs and the physicochemical properties of the interstitial matrix. The above results confirmed that the PPHAuNCs-30-TNCs and PPHAuNCs-50-TNCs efficiently delivered miRNAs into the tumor.

Abraxane is the trade name of paclitaxel (PTX)-loaded human serum albumin (HSA) NPs, which is an example of clinical success of nanomedicine against cancer (Ma and Mumper, 2013). HSA is usually applied to encapsulate certain theraputic drugs to promote their biocompatibility (Desai, 2016). Wang et al. (2016a) constructed a targeted agent by employing PTX and AIEgens for cancer imaging and therapy. The theranostic nanoplatform included four elements: (1) AIEgens conjugated with HSA for imaging; (2) cyclic arginine-glycine-aspartic acid (cRGD)-modified HSA for specific recognition; (3) HSA-functionalized polypyrrole (PPy) for thermotherapy; (4) PTX as a chemotherapy agent and mediating the protein assembly. Firstly, HSA-AIEgens was synthesized by introducing AIEgens into a hydrophobic pocket of HSA, which endowed AIEgens with strong fluorescence. Notably, due to the superb features of AIEgens, the final product exhibited strong fluorescence intensity even after conjugation with quencher-PPy. Next, the imaging performance of the AIEgens-based nanoplatform was investigated to assess tumor treatment efficacy in mice. With intravenous injection for about 1 h, AIEgens fluorescences were observed in the whole bodies of the mice, which were associated with the large amounts of NPs in blood. Thereafter, the nanoagent showed elevated accumulation in tumors with enhanced fluorescence vs. time, which verified its ability to target tumors specifically. Also, the fluorescence of the nanoagent in mice still remained strong after injection for 48 h, which indicated the superior performance of AIEgens in bioimaging and their superb long-lasting retention *in vivo*.

### Persistent Luminescence Imaging

Persistent luminescence is the phenomenon whereby luminescence lasts for several seconds to even days after switching off the excitation source. Generally, two types of active centers, i.e., traps and emitters, contribute to the generation of persistent luminescence. Intrinsic lattice defects or codopants in the host material act as the traps, which are just a few electron volts (eV) below the conduction bands. The emitters are usually lanthanide or transition metal ions. The emergence of persistent luminescence covers four consecutive processes: the formation of charge carriers, trapping of charge carriers, release of the trapped charge carriers, and recombination of the released charge carriers to generate emission.

**Figure 2:**
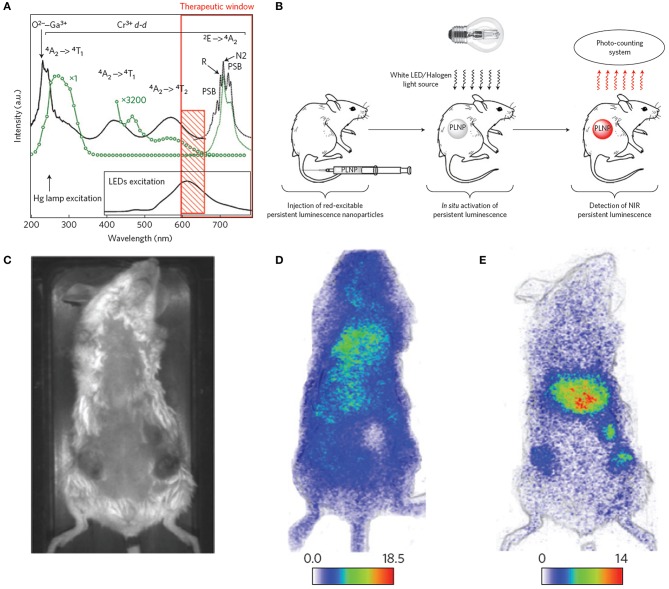
**(A)** Photoluminescence excitation (black solid line) and emission (black dotted line) spectra of Cr^3+^ doped n-ZGO. **(B)** Schematic representation of *in vivo* imaging after *in situ* activation of persistent luminescence nanoparticles (PLNPs). **(C)** Optical image of a tumor-bearing mouse. **(D)** Persistent luminescence image of a tumor-bearing mouse 2 h after injection of ZGO-PEG NPs. **(E)** Persistent luminescence image of a tumor-bearing mouse immediately after LED illumination, 4 h after injection of ZGO-PEG NPs.

ZnGa_2_O_4_:Cr^3+^ is gaining considerable attention because of its strong NIR persistent luminescence upon UV excitation (Bessiere et al., 2011). Maldiney et al. (2014a) introduced the new generation of ZnGa_2_O_4_:Cr^3+^ nanoprobes whose persistent luminescence could be directly charged *in vivo* by incident light with deep penetration and low energy. Low-temperature sintering was adopted to prepare the nanoprobes. The excitation spectrum of n-ZGO photoluminescence showed a broad spectral range from ultraviolet to red light containing four bands (Figure 2A, solid black line). The bands at 245, 290, and 425–560 nm corresponded to an exciton energy higher than the bandgap of ZnGa_2_O_4_, interband excitation, and Cr^3+^ d-d transitions, respectively. The ^4^*A*_2_ → ^4^*T*_2_ absorption band was partly ascribed to weaker absorption of tissue domains (red rectangle, Figure 2A). The n-ZGO PL emission (Figure 2A, dotted black line) consisted of the ^2^*E* → ^4^*A*_2_ bandgap of Cr^3+^. The mechanism for ZnGa_2_O_4_:Cr^3+^ persistent luminescence under visible light was related to antisite defects around the Cr^3+^ ion. Then, ZnGa_2_O_4_:Cr^3+^ nanoprobes were directly injected into mice without preliminary activation (Figure 2B). In the first 2 h, there was no difference in the biodistribution between tumor-bearing and healthy mice (Figures 2C,D). However, persistent luminescence images of tumor-bearing mice clearly showed the tumors 4 h after PEGylated ZGO administration and following visible activation using the orange/red LED source (Figure 2E).

Unfortunately, the persistent luminescence peak (around 700 nm) of ZnGa_2_O_4_:Cr^3+^ made it difficult to use for deep tissue imaging. Also, the emissions of Near-infrared persistent luminescence NPs (NPLNPs) quickly decays over time, leading to lower SNRs. Therefore, superior nanoprobes with higher SNRs are urgently needed for bioimaging. Shi et al. (2018) fabricated a desirable NPLNP, mSiO_2_@Gd_3_-Ga_5_O_12_:Cr^3+^,Nd^3+^ (mSiO_2_@GGO), for bioimaging and cancer treatment. The mSiO_2_@GGO NPs showed an intense emission at 745 nm using 254-nm excitation. The NIR emission was ascribed to the Cr^3+^ spin-allowed ^4^T_2_/^4^A_2_ transition. The 258-nm excitation band was attributed to the GGO host absorption. After the nanoprobes were injected into mice, their luminescence signals were detected throughout the body in 5 min. An intensive luminescence was observed in the liver due to nanoprobes captured by the reticuloendothelial system (RES). The signal intensities of the nanoprobes were even still maintained in the liver after 60 min. This suggested that the nanoprobes could achieve long-duration imaging *in vivo* using their NIR-I luminescence. Also, the mSiO_2_@GGO NPs could realize 2-cm tissue imaging with high SNR (≈5.5). Three main emission peaks were detected at 888, 1,067, and 1,338 nm, corresponding to the eletronic transitions of Nd^3+^ from the excitation level ^4^F_3/2_ to the bottom states ^4^I_9/2_, ^4^I_11/2_, and ^4^I_13/2_ with 808-nm irradiation, respectively. Among them, the emission peak at 1067 nm was predominant, demonstrating high NIR-II emission efficiency. After the nanoprobes were subcutaneously injected into the abdomens of mice, a remarkable NIR-II luminescence signal was detected at the injection site, while negligible intensity was determined at other abdomen areas, suggesting that the nanoprobes can be applied for *in vivo* imaging in the NIR-II region.

PLNPs have gained more attention in deep-tissue bioimaging due to their emission in the NIR region, lack of *in-situ* excitation, and high SNR (de Chermont et al., 2007; Abdukayum et al., 2013). In particular, Cr^3+^-doped PLNPs can produce renewable persistent luminescence under tissue-penetrating LED light, which indicates that the imaging performance of PLNPs is no longer restricted by the light-emitting lifetime (Li et al., 2015a). Chen et al. (2016b) fabricated a novel luminescence probe for bioimaging by combining PLNPs and metallic sulfide. The nanoprobe is attractive due to its ultrasensitive switch-on imaging response to targeted recognition. The PLNP, Zn_1.1_Ga_1.8_Ge_0.1_O_4_:Cr^3+^, served as the light source due to its renewable persistent luminescence. CuS NP is not only as a PTT agent but also serves as a quenching agent for photothermal conversion and NIR absorption. A peptide catalyzed by matrix metalloproteinase (MMP) was introduced to construct the nanoprobe. Further modification with mercapto-PEG and polypeptide endows the nanoprobe with desirable biocompatibility and tumor specificity. To evaluate the activation performance of the nanoprobe, it was intravenously injected into mice after being treated by UV light for 10 min. Remarkable luminescence was clearly detected at the tumor site of mice for about 2 h, showing efficient MMP activation and the superior tumor-targeting recognition of the probe.

Some PLNPs consisting of host nanomaterials and codopants have been extensively investigated; please see details in Table 1 (Liu et al., 2019a).

**Table 1:** Persistent luminescence NPs for bio-imaging and therapy.

**Hosts**	**Dopants**	**Comments and applications in bio-imaging**	**References**
Gd_2_O_2_S	Eu^3+^,Mg^2+^,Ti^4+^	Regular NP shape, bimodality optical/MRI	Rosticher et al., 2015
Ca_3_(PO_4_)_2_/hydroxyapatite	Mn^2+^,Tb^3+^,Dy^3+^	Fully biocompatible, NPs and *in vivo* imaging	Rosticher et al., 2015
Ca_2_Si_5_N_8_	Eu^2+^,Tm^3+^	Bioimaging applications	Maldiney et al., 2012
SrAl_2_O_4_	Eu^2+^,Dy^3+^	NPs, functionalization, Bioimaging applications, green emission	Zeng et al., 2018
Ca_0.2_Zn_0.9_Mg_0.9_Si_2_O_6_	Mn^2+^,Eu^2+^,Dy^3+^	NPs, functionalization, pioneer work for bio-imaging: cancer cells imaging, cell targeting	de Chermont et al., 2007
Ca_1.86_Mg_0.14_ZnSi_2_O_7_	Eu^2+^,Dy^3+^	FRET and various bio-sensing applications	Sun et al., 2018
CaMgSi_2_O_6_	Mn^2+^,Eu^2+^,Pr^3+^	NPs, functionalization, bio-imaging	Maldiney et al., 2011
MAlO_3_ (M = La, Gd)	Mn^4+^/Ge^4+^	Bio-imaging in pork tissue	Liu et al., 2016
GdAlO_3_	Mn^4+^,Ge^4+^Au Sm^3+^,Cr^3+^	Trimodality imaging Optical and magnetic dual mode imaging	Liu et al., 2016 Li et al., 2018b
ZnGa_2_O_4_	Cr^3+^	NPs, functionalization, bio-imaging (cancer cells imaging), Cell targeting, cytotoxicity, visible Light NIR photostimulation X-rays activation Oral administration Breast cancer imaging Toxicology analysis Protobiotic analysis	Maldiney et al., 2014a Xue et al., 2017; Liu et al., 2018a Ramirez-Garcia et al., 2017; Liu et al., 2017
ZnGa_2_O_4_ in hollow cavity	Cr^3+^	Photodynamic therapies	Wang et al., 2018
ZnGa_2_O_4_	Cr^3+^,Gd^3+^	NPs, functionalization, bimodality optical/NMR imaging	Maldiney et al., 2015
ZnGa_2_O_4_/SiO_2_	Cr^3+^	Core-shell structure, drug delivery	Maldiney et al., 2014b
ZnGa_2_O_4_/Fe_2_O_3_	Cr^3+^	Cell labeling and magnetic vectorization	Teston et al., 2018
ZGOCS@m-SiO_2_@Gd_2_O_3_	Cr^3+^	Multimodal nanoprobes	Zou et al., 2017
Zn_1.1_Ga_1.8_Ge_0.1_O_4_/SiO_2_	Cr^3+^,Eu^3+^	NPs, core-shell structure, drug delivery	Shi et al., 2015a
Zn_3_Ga_2_Ge_2_O_10_	Cr^3+^	Imaging of pork tissue, Photostimulation, cytotoxicity	Li et al., 2014a
Zn_1.1_Ga_1.8_Ge_0.1_O_4_@SiO_2_	Cr^3+^	Bio-imaging and drug delivery	Liu et al., 2018b
Zn_1.25_Ga_1.5_Ge_0.25_O_4_	Cr^3+^,Yb^3+^,Er^3+^	Metastasis tracking and chemo-photodynamic therapy	Li et al., 2018c
Zn_1.1_Ga_1.8_Ge_0.1_O_4_	Cr^3+^	Nanothermometry	Yang et al., 2017a
Zn_3_Ga_2_Sn_1_O_10_	Cr^3+^	Imaging of goldfish	Li et al., 2014b
Zn_2.94_Ga_1.96_Ge_2_O_10_	Cr^3+^,Pr^3+^	NPs, functionalization	Abdukayum et al., 2013
Zn_3_Ga_2_Ge_2_O_10_	Cr^3+^	Recognition of breast cancer cells	Li et al., 2015b
Zn_3_Ga_2_GeO_8_	Cr^3+^,Yb^3+^,Er^3+^	Upconversion	Liu et al., 2014b
LiGa_5_O_8_	Cr^3+^/PEG-OCH_3_	NPs, functionalization, bio-imaging, Visible light stimulation, photostimulation	Liu et al., 2013; Fu et al., 2014
Ca_3_Ga_2_Ge_3_O_12_	Cr^3+^,Yb^3+^,Tm^3+^ Pr^3+^,Yb^3+^	NIR stimulation, upconversion *in vivo* imaging	Chen et al., 2014; Dai et al., 2017
m-SiO_2_@Gd_3_Ga_5_O_12_	Cr^3+^,Nd^3+^	Multimodal imaging and cancer therapy	Shi et al., 2018
Sr_2_SnO_4_	Nd^3+^	Finger image	Kamimura et al., 2014
SiO_2_/CaMgSi_2_O_6_	Eu^2+^,Pr^3+^,Mn^2+^	Bio-imaging, intraperitoneal injection Photostimulation imaging of pork tissue	Li et al., 2014c
Y_3_Al_2_Ga_3_O_12_	Er^3+^,Cr^3+^	Imaging in the second biological window	Xu et al., 2018b
NaYF_4_ + SrAl_2_O_4_	Yb^3+^,Tm^3+^, Eu^2+^,Dy^3+^	Upconversion & photodynamic therapy	Hu et al., 2018b
Sr_2_MgSi_2_O_7_	Eu^2+^/^3+^,Dy^3+^	Photodynamic activation Visualization of abdominal inflammation	Homayoni et al., 2016 Yu et al., 2018
La_3_Ga_5_GeO_14_@SiO_2_@Van(vancomycin)	Cr^3+^,Zn^2+^	Bio-imaging-guided *in vivo* & drug delivery	Zhan et al., 2018
CaTiO_3_	Pr^3+^,Yb^3+^,Tm^3+^	Upconverting and guided photothermal therapy	Zhao et al., 2017
ZnSn_2_O_4_	Cr^3+^,Eu^3+^	Cellular and deep tissue imaging	Li et al., 2017a
Sr_3_Sn_2_O_7_	Nd^3+^	Second window imaging	Kamimura et al., 2017

### Near-Infrared Surface-Enhanced Raman Scattering (NIR SERS) Imaging

NIR optical nanoprobes have received increasing attention as multimodal therapeutic reagents for bioimaging and photothermal tumor elimination. For optical imaging, SERS has emerged as an attractive method with intense signal response and good specificity (Gandra and Singamaneni, 2013; Zhang et al., 2018). A narrow bandwidth, high resistance to photobleaching, and autofluorescence make SERS an effective tool for non-invasive single-cell assay, rapid disease diagnosis, and nanomedical imaging (Maiti et al., 2012; Guerrini et al., 2017). Three parts, including a noble metal matrix, Raman active reporters, and a biocompatible surface coating, constitute a typical NIR SERS nanoprobe.

**Figure 3:**
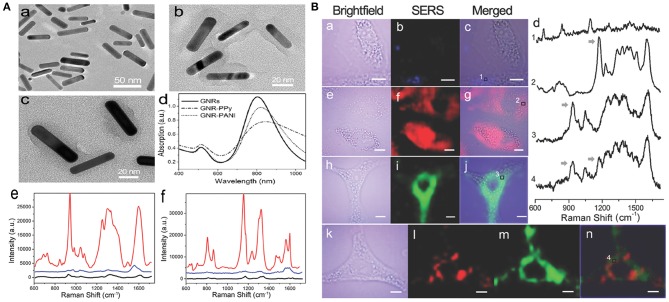
**(A)** Characterization of SERS nanoprobes: (a–c) TEM images of GNRs, GNR-PANI, and GNR-PPy, respectively. (d) UV-vis-NIR absorbance spectra of the nanostructures. Raman spectra of GNR-PPy (e) and GNR-PANI (f) under 785 nm (red line) and 514.5 nm (blue line) laser excitation, respectively. Black lines show the normal Raman spectra of CP molecules. **(B)** NIR SERS imaging of A549 cells after incubation with GNRs (a–c), GNR-PANI (e–g), and GNR-PPy (h–j) for 4 h. (d) Raman spectra in the cytoplasmic compartments. Arrows indicate the Raman peaks used for SERS imaging. (k–n) Two-color imaging after incubation with a mixture of two tags [brightfield image (k), GNR-PANI (l), GNR-PPy (m), merged image (n)].

The NIR SERS probe is usually prepared through direct modification of a reporter molecule (e.g., a dye) onto the metal surface. However, the electrostatic force-based metal-dye framework leads to structural instability, particularly in physiological environments (Wang et al., 2013b). Strong cytotoxicity is another defect that limits the uses of dye-based SERS probes in nanomedicine. Outer covering with hydrophilic thiolated polyethylene glycol (PEG-SH) can evidently improve the biocompatibility of the metal-dye system. However, competitive binding of polymer and dye reporter with the noble metal can reduce the SERS signal intensity of probes. Also, this kind of PEG-based modification is unsuitable for PTT owing to its labile steric conformation, which is subject to morphological change (melting) upon high-power NIR irradiation (Wang et al., 2012). Liu et al. (2015d) developed environmentally friendly NIR SERS nanoprobes instead of using highly toxic organic dyes. Multifunctional conducting polymer (CP), acting as an outer modification layer and Raman-active molecule, was immobilized on the surface of gold nanorods (GNRs). The GNR-CP nanostructure was separately fabricated through oxidatively chemical polymerization, utilizing two different combinations of monomer and oxidant, namely pyrrole/FeCl_3_ and aniline/(NH_4_)_2_S_2_O_8_. Rod-shaped GNRs were synthesized with average dimensions of 42.3 ± 6.9 nm long and 9.6 ± 1.4 nm wide (Figure 3A). Such nanoparticles are considered to have an EPR effect and long-term retention in tumors. The UV-vis-NIR absorption spectra of GNRs showed a transverse and a longitudinal SPR peak at around 515 and 800 nm, respectively. Figure 3Ab,c show different constructions of two probes, GNR-polyaniline(PANI) and GNR-polypyrrole(PPy). Remarkable shifts toward longer wavelengths and reductions in absorbances were observed in the absorption curves of GNR-CPs (Figure 3Ad). The SERS fingerprints of the nanoprobes were dependent on excitation light source. Negligible SERS signal were detected as the probes were illuminated with 514.5 nm light. In comparison, very strong SERS signals appeared upon 785 nm excitation. The strongest peaks were located at 945 cm^−1^ for GNR-PPy and 1,170 cm^−1^ for GNR-PANI (Figure 3Ae,f), which were ascribed to in-plane distortion and bending vibrations of C-H in the quinoid group, respectively.

A549 cells exposed with the probe were investigated by utilizing the above NIR SERS imaging. After cell exposure to GNRs, the SERS images exhibited a very weak response from cellular elements in the 600–1,700 cm^−1^ range (Figure 3Ba–c). However, distinct SERS signals were obtained from cells exposed to GNR-PANI (Figure 3Be–g) or GNR-Ppy (Figure 3Bh–j). The results demonstrated that the probes were mainly distributed in the cytoplasm. The characteristic signals from the cytoplasm were detected clearly in the SERS spectra (Figure 3Bd), displaying the availability of SERS probes in the sophisticated cellular environment. Moreover, cancer cells were cotreated with the two probes. Figure 3Bl,m show SERS images of the probes and the individual response distribution in cells. Notably, each mapping was separate, which was attributed to the narrow bandwidth of the Raman peaks.

Silver bumpy nanoshell (AgNS) has been explored as a highly active NIR SERS nanoprobe for the biomedical field (Premasiri et al., 2018). AgNS has intense scattering in the NIR-region, where its SERS enhancement factor is higher than those of gold nanorods or nanospheres. Also, AgNS has strong NIR absorption, which is useful for PA imaging. Additionally, AgNS do not induce any *in vivo* toxicity, as confirmed by cytotoxicity tests (Baumberg et al., 2017). Cha et al. (2017) demonstrated dual-modal detection of sentinel lymph nodes (SLNs) using a silica-coated silver bumpy nanoshell probe (AgNS@SiO_2_): imaging the SLN with a PA signal and *in vivo* multiplex identification of targets in the located region with SERS. Using the correctional Stöber approach, the surface of AgNS was covered by silica-shell and finally treated with bovine serum albumin (BSA) to enhance biocompatibility. AgNS@SiO_2_ possesses some advantages, e.g., high dispersion stability, facile surface modification, and good biocompatibility. PA imaging was adopted to detect the accumulated regions of AgNS@SiO_2_, and the SERS signals were analyzed to identify different ratios of several kinds of Raman-labeled AgNS@SiO_2_. After three different types of Raman-labeled AgNS@SiO_2_ probes were injected into rat's left front paw pad, respectively, their PA and SERS signals were monitored and analyzed to locate the SLN and identify the targeted AgNS@SiO_2_. They successfully obtained PA images and SERS spectra using the portable-Raman system with a 785-nm NIR-excited laser. This dual-modal imaging system may be utilized in diagnostic fields, e.g., multiplex cancer marker detection *in vivo*.

### Photoacoustic (PA) Imaging

Photoacoustic (PA) imaging, a new method of visualization via photoacoustic response, has increasing potential in nanomedical fields (Kim et al., 2010). PA imaging has been explored to visualize biostructures from organelles to cells to organs (Wang and Hu, 2012). A contrast agent absorbs the excitation energy and converts it into thermal energy. Thus, a wideband ultrasound emission is produced due to heat-induced transient thermoelastic expansion, which is collected with an acoustic detector and transformed into PA images. PA imaging combines the good specificity of optics and the deep-tissue transmission of ultrasound (US), which overcomes the restrictions of traditional imaging (Nie and Chen, 2014).

**Figure 4:**
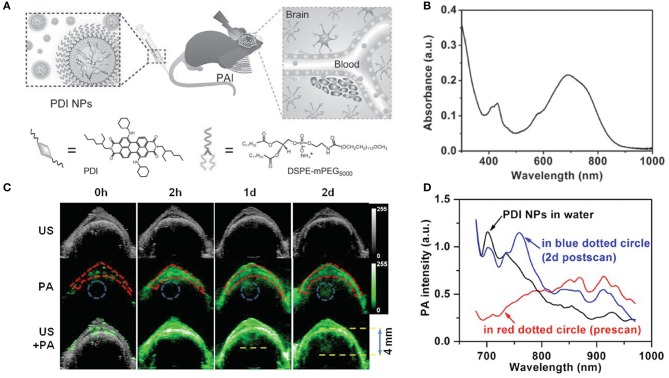
**(A)** Schematic illustration of the PA imaging process of brain tumor *in vivo* by PDI NPs. **(B)** UV-vis-NIR absorption spectrum of PDI NPs in aqueous solution. **(C)** Ultrasonic (gray), photoacoustic (green), and their overlay brain coronal sections of the tumor model after tail vein injection of PDI NPs. **(D)** PA spectra of PDI NPs in different media. Skull region in the red dotted circle of **(C)** before NP injection (red line) and tumor region in the blue dotted circle of **(C)** after 2 d NP injection (blue line).

Perylene-3,4,9,10-tetracarboxylic diimide (PDI) and its derivatives have been extensively applied to fabricate diverse electronic devices based on their extraordinary physicochemical properties, easy functionalization, and extremely low price (Perrin and Hudhomme, 2011; Birel, 2017). Fan et al. (2015) constructed an efficient PDI-based NIR-absorptive contrast nanoagent enveloped by micelle for PA imaging of deep brain tumor in living mice. To make PDI absorb in the NIR-region, tertiary amine and diimide as donor and acceptor were introduced to fabricate a classical donor-π-acceptor system for increasing redshift efficacy. Water-soluble PDI NPs were prepared by wrapping with amphiphilic PEG derivatives (Figure 4A). These PDI NPs displayed strong NIR absorption at around 700 nm in aqueous solution (Figure 4B). After 2-h injection, the PA signal at the skull region (red dotted circle) intensified greatly due to the circulation of PDI NPs in the blood vessel. After 1-d injection, a significant PA signal appeared in the brain tumor region (blue dotted circle) of mice, but no signal was found in the control group. Also, a 4.0-mm depth of the tumor region was more clearly observed after 2-d injection compared with a shallower depth (~3.0 mm) after 1-d injection (Figure 4C), which was attributed to the enhanced tumor penetration depth of NPs with time. The biodistribution of PDI NPs in brain tumor was verified by measuring PA spectra. Strong PA intensities at 700 and 735 nm (blue line) were clearly observed in the tumor region after 2-d NP injection (Figure 4D), which was consistent with the NIR absorption peak of PDI NPs (Figure 4B). This indicated the successful accumulation of PDI NPs in the tumor.

Carbon dots (CDs) are promising carbon-based imaging probes due to their excellent biocompatibility, water solubility, and photostability (Yang et al., 2009; Li et al., 2012, 2013). However, the main challenge of CDs for practical application is guaranteeing their biodegradability (Tang et al., 2013; Ge et al., 2015; Jiang et al., 2015). Moreover, the mechanisms of the interaction between CDs and low-energy photons are being investigated to obtain high resolution and contrast for CD optical imaging. Lee et al. (2016) synthesized a biocompatible and N-doped type of CD (N-CDs) for PA imaging. N-CDs were prepared by solvothermal carbonation using citric acid and HNO_3_ as a C and N source. N-CDs showed strong absorption in the NIR region (680–800 nm). N-CDs with high contents of N atoms raised the ambient temperature more quickly upon NIR (680–808 nm) laser irradiation. This was because a high N content produced more bandgaps so that the carrier could be captured from LUMO to a bandgap and relaxed between bandgaps. N-CDs exhibited higher heat conversion efficiency than conventional PA contrast agents (e.g., GNR and methylene blue) under the same optical density. Therefore, N-CDs could produce sufficiently intense PA effects to realize non-invasive imaging and thermal therapy *in vivo*. The researchers carried out time-resolved PA imaging of SLNs of Sprague-Dawley rats and evaluated the biodegradability of N-CDs through renal clearance. The relative PA signal was suddenly enhanced in SLNs 30 min after hypodermic injection of N-CDs and reduced gradually until 180 min. Meanwhile, the PA signal from the bladder area rose intensively at 100 min, suggesting the effective removal of N-CDs to the urine. For tumor PTT, the N-CDs displayed complete tumor ablation without recurrence for Balb/c nude xenograft HepG2 tumor model mice with a tumor volume of 27 mm^3^ upon 808-nm NIR laser irradiation. N-CDs, as a kind of NIR-absorbing nanomaterial, will become a potent contrast agent for PA bioimaging and for improving PTT efficacy.

A new type of optical nanoagent, semiconducting polymer NP (SPNP), provides an inspiring strategy for solving the nanotoxicity problem (Li and Pu, 2019). SPNPs with highly conjugated structures exhibit excellent photothermal conversion efficiency and photostability (Li et al., 2018d). Moreover, the molecular versatility of SPNPs is not only beneficial to facile tuning of the spectrum independent of size or morphology but also allows the skeleton or side chain to be easily modified for on-demand functionalization (Feng et al., 2013). Based on these merits, SPNPs have been used as promising PA agents for biomarkers and cancer detection (Lyu and Pu, 2017). Furthermore, preliminary investigation has revealed that SPNPs have the advantage of being degradable NIR-I nanoagents (Lyu et al., 2018). Nevertheless, whether they can perform as biodegradable NIR-II nanoagents has not been explored. Jiang et al. (2019) fabricated metabolizable NIR-II SPNP-based agents for PA imaging. By introducing the strong electron-withdrawing group benzobisthiadiazole, the synthesized SPNPs (SPNP-PT, SPNP-DT, and SPNP-OT) showed strong absorption at 1,079 nm and superior photothermal conversion efficiencies at 1,064 nm. Owing to the oxidizable thiophene group and hydrolyzable PEG-based matrix in their skeletons, three NIR-II SPNPs (~30 nm) could be effectively decomposed into smaller sizes NIR NPs (~1 nm) by myeloperoxidase or lipase with high abundance *in vivo*. Further, the SPNPs were completely eliminated in 15 days through renal or hepatobiliary metabolism in living mice. Especially, SPNP-PT demonstrated good SNRs for PA imaging of tumor or brain vasculature in mice under a systematic dosage (2.5 mg kg^−1^) lower than that of other reported agents (≥10 mg kg^−1^).

Overall, various nanomaterials have been exquisitely designed for deep-tissue PA imaging. PA contrast agents are mainly categorized into inorganic and organic nanoagents based on their structures (Table 2) (Wang et al., 2016b).

**Table 2:** Examples of PA contrast agent explored in PA imaging.

**Materials**		**Types of nanoagents**	**Advantages (+)/Disadvantages (-)**	**References**
Inorganic	Metallic nanomaterials	Au nanorods; Au nanostars; Au nanocages; Au nanoshell; Au nanovesicles; Au nanoflowers; Ag nanoplates; Palladium nanoplates; antimony nanoparticles	(+) tunable physiochemical properties; chemically inert element with reasonable biocompatibility; able to carry cargoes. (–) non-biodegradability; suboptimal photothermal stability	Chen et al., 2015b; Zhong et al., 2015 Wang et al., 2015a Zhang et al., 2013 Topete et al., 2014 Song et al., 2015a Huang et al., 2014a Homan et al., 2012 Nie et al., 2014 Li and Chen, 2015
	Carbon-based nanomaterials	Carbon nanotubes; Graphenes; Carbon dots	(+) able to carry cargoes; good photothermal stability. (–) non-biodegradability; heterogeneity	Zhang et al., 2015 Lalwani et al., 2013; Sheng et al., 2013; Ge et al., 2015
	Transition metal chalcogenides (TMC)-based nanomaterials	CuS; WS_2_; MoS_2_; FeS; Bi_2_S_3_; CuSe; Co_9_Se_8_; Bi_2_Se_3_	(+) high photothermal conversion efficiency; good photothermal stability; low cost. (–) non-biodegradability; contain heavy metal elements	Cheng et al., 2014a; Yin et al., 2014 Cui et al., 2015; Yang et al., 2015b Hessel et al., 2011; Liu et al., 2015e Song et al., 2015b
Organic	Dyes	Porphyrin- and Cyanine-based dyes, e.g., Indocyanine green (ICG), IR780, IR825, etc.	(+) good biocompatibility/biodegradability. (–) poor aqueous solubility, low photothermal stability, short bloodstream circulation half-life	Sheng et al., 2014; Wang et al., 2015b Lovell et al., 2011; Song et al., 2015c Huang et al., 2014b; Chen et al., 2015a; Rong et al., 2015
	Polymer-based nanomaterials	Polypyrrole; Polyaniline; Polydopamine; Semiconducting polymers	(+) good biocompatibility and photothermal stability; able to carry cargoes. (–) their biodegradation behaviors remain unknown	Yang et al., 2011; Lin et al., 2014 Yang et al., 2012; Pu et al., 2015

## Optically Active Nanomedicine for Targeted Therapy and Drug Delivery

### Photodynamic Therapy (PDT)

Photodynamic therapy (PDT) employs photosensitizers (PSs) and light of a specific wavelength in combination with molecular oxygen to generate reactive oxygen species (ROS) that kill cancer cells through the oxidation of important biomolecules and organelles (Zhou et al., 2016a). The introduction of NPs in PSs provides the following advantages: (1) effective PS delivery to the target site, (2) easy phase transfer of hydrophobic PSs into an amphiphilic bloodstream to increase circulation time, (3) use of the EPR effect for the effective diffusion of PSs into tumors, (4) surface modification with various molecules, enhancing the cellular uptake and targeting, and (5) multiple functionality by combining the properties of NPs (e.g., multi-modal imaging) with those of PSs (Lucky et al., 2015). A myriad of inorganic and organic nanostructured PSs, e.g., gold NPs (Dykman and Khlebtsov, 2012), metallic oxides (Bechet et al., 2008), carbon-based materials (Albert and Hsu, 2016), mesoporous silica (Montalti et al., 2014), polymeric micelles (Elsabahy et al., 2015), and UCNPs (Wang et al., 2017), have been developed for image-guided PDT therapies (Lan et al., 2019).

**Figure 5:**
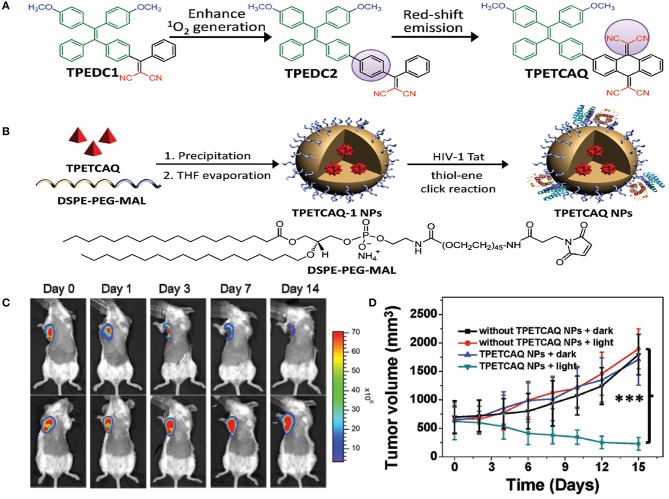
**(A)** Design of TPETCAQ. **(B)** Synthesis of TPETCAQ-1 NPs and TPETCAQ NPs. **(C)** Time-dependent bioluminescent 4T1-luc tumor imaging of mice after intratumoral administration of TPETCAQ NPs (30 μL, 1 mg mL^−1^ for TPETCAQ, top panel) or saline (bottom panel) with light irradiation (300 mW cm^−2^, 5 min) at 1 h post-injection. **(D)** Tumor volume measurement for different groups of mice. ^***^P < 0.001.

Recently, AIE PS, a new family of PSs, has been gaining more attention (Hu et al., 2018a). Owing to the inhibition of non-radiative energy consumption and RIM, AIE PSs exhibit enhanced signal intensity (Alifu et al., 2017) and produce more ROS species in the aggregate state (Gu et al., 2017). These properties of AIE-based PSs make them a better choice for image-guided PDT and tumor-killing (Wu et al., 2017a). However, PSs with NIR emissions and high ROS production still remain to be explored. Wu et al. (2017b) reported the fabrication of a kind of PS TPETCAQ (Figure 5A) with AIE characteristics for NIR image-guided PDT. Encapsulation of TPETCAQ using a DSPE-PEG-MAL matrix and subsequent surface modification with the HIV-1 transactivator were used to obtain the final product, TPETCAQ NPs (Figure 5B). The resultant product exhibited strong fluorescence emission intensity at 820 nm, accompanied by much more ^1^O_2_ production than Ce6, a well-known highly efficient PSs. The bioluminescence of tumor *in vivo* progressively reduced with time, which demonstrated that the nano-PSs were efficient for image-guided PDT. Also, only very faint luminescence signals were detected from the AIE-based PSs after 14 d, suggesting their high PDT efficacy (Figure 5C). The excellent PDT efficacy of the AIE PSs was further confirmed by tumor volume changes for different groups (Figure 5D).

In addition to low toxicity, photobleaching resistance, and deep tissue penetration, UCNPs also have the unique properties of transforming from NIR emission to visible light (Gu et al., 2013; Chan et al., 2015). Notably, the long-lived red emissions from UCNPs overlap partly with the absorptions of some PSs, e.g., ZnPc or Ce6, which offers a valid energy transfer pathway to activate PSs for PDT under NIR irradiation (Idris et al., 2015; Tian et al., 2015). Huang et al. (2016) prepared Na_0.52_YbF_3.52_:Er UCNPs with intense red emission using a simple solvothermal approach for multimodal imaging and tumor PDT. A low [Na]/[Yb] ratio in UNCPs was found, owing to the lack of elemental Na, which resulted in high luminescence intensity and color purity for red UC emission of Er^3+^. The UNCPs displayed dominant red and weak green UC emission at 655 and 522/543 nm, which were attributed to the intra-4f transitions of Er^3+^, ^4^*F*_9/2_ → ^4^*I*_15/2_, and ^2^*H*_11/2_/^4^*S*_3/2_ → ^4^*I*_15/2_, respectively. In addition, a thin SrF_2_ layer was modified on the UNCPs, and the red UC emission intensity was increased by about 17 times, while a relatively high intensity ratio (~5.8) of Red/Green was still retained. To make them utilizable *in vivo*, the Na_0.52_YbF_3.52_:Er@SrF_2_ UCNPs were further modified with DSPE-PEG to obtain the final low toxicity product Lipo-UCNPs. Significantly, the PS ZnPc-loaded Lipo-UCNPs exhibited high efficacy in ^1^O_2_ production and cancer cell killing under 915 nm irradiation, avoiding the overheating effect usually resulting from 980 nm excitation. After intratumoral injection of 50 μL ZnPc-Lipo-UCNPs (10 mg mL^−1^) into HeLa tumor-bearing mice, the tumor volumes increased much more slowly, demonstrating the significant tumor growth rate inhibition effect of the UNCP-based PDT. The results revealed the great potential of UCNPs with high-purity red emission for imaging-guided PDT of tumors.

Most PSs for PDT possess poor solubility in water and suboptimal selectivity *in vivo* (Yan et al., 2015). To overcome these limitations, PSs are usually conjugated with nanocarriers with a high surface area, e.g., GO (Yang et al., 2013). Various PSs have been designed to load on the surface of GO via π-π stacking and hydrophobic forces for tumor imaging and PDT in animal models (Rong et al., 2014; Shi et al., 2014). However, most PS-GO nanoconjugates passively targeted to tumors *in vivo via* an EPR effect. Thereby, some tumor-specific molecules (e.g., peptides, ligands, and antibodies) need to be modified onto GO to increase targeting and PDT efficacy (Shi et al., 2015b). Yu et al. (2017) fabricated a PS-loaded GO nanocomplex by conjugating PEGylated GO with a tumor-selective HK peptide that can specifically bind to highly expressed integrin αvβ6 receptor in many tumor types and following functionalization with a PS. The tumor uptake of GO(PS)-PEG-HK was obviously higher than that of free PS and GO(PS)-PEG, demonstrating the specific intake of the nanocomplex in tumors by the recognition of integrin αvβ6. Tumor recurrence, especially lung metastasis, was significantly inhibited in mice for nearly a month by vaccination with necrotic 4T1 tumor cells induced by the nanocomplex PDT. The nanocomplex-treated necrotic tumor cells could activate dendritic cells and significantly inhibit tumor growth and lung metastasis by increasing the infiltration of cytotoxic CD8^+^ T lymphocytes in tumor. Using the nanocomplex, the primary tumor, and the residual tumor cells could be efficiently killed by activating host anti-tumor immunity and stimulating the immune memory, accordingly prohibiting distant metastasis. These findings suggest that nanocomplex-based PDT is an effective strategy for eliminating residual cells after tumor resection and preventing tumor recurrence and distant metastasis.

### Photothermal Therapy (PTT)

Photothermal therapy (PTT) utilizes the photothermal effect of a photothermal transduction agent (PTA) that can harvest the energy from light and transform that energy into heat to raise the surrounding temperature and induce the death of cancer cells. An ideal PTA should possess high photothermal conversion efficiency (PCE), strong absorption in the NIR region, and good accumulation in tumors without toxic side effects. In particular, nano PTAs can accumulate in tumors through the EPR effect and active targeting. Also, nano PTAs can realize higher PCE than small-molecule PTAs and can potentially be used with multiple imaging modalities and incorporate various therapeutic functions for advanced application (Cheng et al., 2014b). Nano PTAs can be divided into inorganic and organic materials (Gai et al., 2018). Inorganic materials include noble metals (Riley and Day, 2017), metal chalcogenides (Li et al., 2017b), carbon-based materials (Hong et al., 2015), and other two-dimensional (2D) materials (e.g., black phosphorus, nanosheets, boron nitride, graphitic carbon nitride, MXenes) (Chen et al., 2015c; Augustine et al., 2017; Tan et al., 2017; Choi et al., 2018; Huang et al., 2018). Organic PTAs include semiconducting polymer NPs (SPNPs), nanomicelle-encapsulated NIR dyes, and porphysomes (Jung et al., 2018; Liu et al., 2019b).

**Figure 6:**
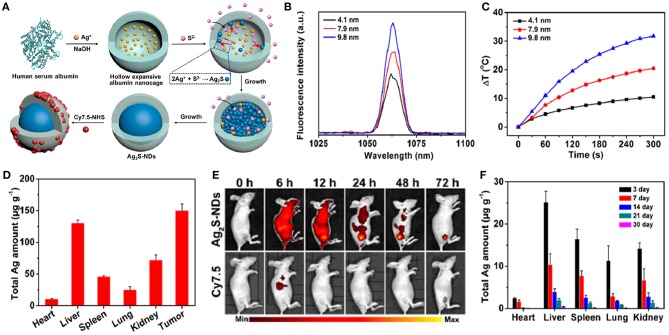
**(A)** Schematic illustration of construction of theranostic Ag_2_S nanodots in HSA Nanocages. **(B)** Fluorescence spectra of Ag_2_S-NDs. **(C)** Temperature elevations of Ag_2_S-NDs under 5 min irradiation (785 nm, 1.5 W cm^−2^). **(D)** Biodistribution of Ag_2_S-NDs at 24 h post-injection. **(E)** NIR fluorescence imaging of 4T1-tumor-bearing mice treated with Cy7.5-labeled Ag_2_S-NDs at 72 h post-injection. **(F)** Long-term distributions of Ag_2_S-NDs in various major tissues during 30 days post-injection. Dose: 50.0 μmol kg^−1^ Ag.

Recently, stoichiometric semiconductor metal sulfide nanocrystals (e.g., Ag_2_S and CuS) have been developed for optical imaging and PTT due to their strong absorption capacity in the NIR-region, negligible photobleaching, high photoconversion efficiency, ultrasmall size, and good inertia (e.g., the solubility product constant is *K*_sp_ = 6.3 × 10^−50^ for Ag_2_S) (Hong et al., 2012b). Yang et al. (2017b) designed dimension-dependent Ag_2_S nanodots (NDs) as a photothermal agent for PTT and multimodal imaging. These NDs are prepared by finely modulating their growth within clinically acceptable HSA nanocages (Figure 6A). They exhibited a narrow emission band (full-width at half-maximum ~8.0 nm) due to their monodisperse size distribution (Figure 6B). Also, larger NDs showed a higher fluorescence response in the NIR-II range, suggesting that the fluorescence spectrum strongly depended upon their diameters. Ag_2_S-NDs also displayed obvious size-dependent temperature elevations (Figure 6C), mainly originating from their size-dependent molar extinction coefficients. Additionally, strong non-radiative energy decay in the conduction band may contribute to the efficient photothermal conversion efficacy of the NDs. The improved circulation and EPR effect of NDs caused them to be mainly distributed into tumor, suggesting their high uptake in tumor (Figure 6D). The NDs showed remarkable fluorescence within tumor at 48 h after injection, whereas the control group displayed a faint signal due to fast elimination of NDs (Figure 6E). These findings indicated that the NDs could be accumulated at the tumor site for a long time, which was beneficial to flexible imaging or PTT. The NDs showed time-dependent uptakes in other tissues, including heart, liver, spleen, lung, and kidney. Moreover, the NDs were progressively excluded from the tissues in 30 days via renal excretion (Figure 6F), thus avoiding potential toxicity concerns.

Copper chalcogenide (Cu_2−n_R, R = S, Se, Te, 0 ≤ n ≤ 1) NPs, which possess strong localized SPRs (LSPRs) in the NIR region due to copper deficiency, have been employed for PA imaging and PTT (Coughlan et al., 2017). Cu_2−n_R NPs may be much smaller than conventional photothermal agents such as GNPs (typically >50 nm in 1D to generate NIR LSPR). Also, their good biodegradability allows the release of the vital trace elements copper and chalcogen to maintain the health of the organism (for example, selenium or copper deficiency contributes to the incidence and mortality of some cancers [e.g., liver, prostate, and lung)] or the evolution and aggravation of some cardiovascular diseases and diabetes (Zhou et al., 2016b). Monodisperse Cu_2−n_Se NPs are usually synthesized in organic solvents at high temperature, and they then need laboriously subsequent modification to make them utilizable in biological fields (van der Stam et al., 2015; Yan et al., 2017). Zhang et al. (2016) prepared novel ultrasmall PEGylated Cu_2−n_Se NP in aqueous solution for multimodal imaging-guided tumor PTT. The Cu_2−n_Se NPs were synthesized in distilled water and formed by a characteristic dark green solution and were then functionalized with dimercaptosylated PEG to enhance their solubility, dispersity, and biocompatibility after ultrafiltration. The final product displayed strong LSPRs in the NIR region (600–1,100 nm) owing to the high hole density due to copper deficiency. The high extinction coefficient (8.5 Lg^−1^ cm^−1^) of the PEGylated Cu_2−n_Se NPs at 808 nm reflected a distinct photothermal conversion capability. Also, the NPs showed good photothermal stability and long-term circulation with a half-life of 8.14 h owing to the presence of water-soluble and large sterically hindered PEG. After intravenous injection of the PEGylated Cu_2−n_Se NPs into BALB/c mice bearing 4T1 tumors (130 mm^3^ volume), the tumor temperature of the mice rose steeply to 57.6°C during NIR irradiation, which was high enough to kill the cancer cells and to halt their lethal proliferation. The tumors shrank and became black scars at day 3 and were removed totally in 16 d without recurrence. These NPs might become an effective PTT agent for *in vivo* tumor therapy.

Nanodiamonds (NDDs) with diameters of 2–10 nm and a truncated octahedral framework have appeared as innovative materials for bioimaging and therapy due to their good biocompatibility, spherical morphology, high density, large surface area, and surface functionality (Mochalin et al., 2011). Ryu et al. (2016) designed folic acid (FA)-conjugated NDD (FA-NDD) nanoclusters by using the unique features of NDDs for PTT. FA was selected as a model targeting ligand for tumor and receptor-mediated endocytosis since its receptors are generally overexpressed in some types of tumor cells. Their spherical morphology and good biocompatibility enabled NDDs to act as a nanoplatform for delivery systems. Amination of NDD nanoclusters with -COOH via 1,2-ethylenediamine and subsequent modification with FA by carbodiimide chemistry were performed. After 5-min laser irradiation, the temperature of NDD (10 μg mL^−1^) rose to 54°C. Compared with WI-38 cells (negative control), FA-NDD nanoclusters easily entered the KB cells (positive control), indicating specificity to tumor cells that overexpress FA receptors. Cell viability assay and fluorescence microscopic imaging clearly showed that FA-NDD preferred to ablate KB cells selectively rather than WI-38 cells under NIR laser illumination. For tumor-bearing nude mice, a substantial accumulation of FA-NDD in tumor led to a significant decrease in tumor volume, and almost absent 14 d after NIR laser exposure as compared to NDD nanoclusters. These results clearly verified that the combination of the FA-NDD nanoclusters and NIR light might be an efficient and feasible tumor therapy.

### Optical-Responsive Drug Delivery

The effective delivery and release of drugs to targets remains a great challenge to improving therapies for human diseases (Devadasu et al., 2013). A recognized strategy is to construct a target-specific drug delivery system (DDS) that can carry an efficient dosage of the drug to targeted cells and tissues (Wong and Choi, 2015). An ideal stimuli-responsive DDS should possess the following features: biocompatible or biodegradable composition, high drug-loading capacity, a site-specific delivery mechanism to spare normal cells and tissues, no premature drug release, and accurate release in response to exogenous or endogenous stimulus (Karimi et al., 2016; Liang et al., 2016). Various nanocarriers such as liposomes (Grimaldi et al., 2016), polymers (Nicolas et al., 2013; Tong et al., 2014), micelles (Cabral et al., 2018), dendrimers (Astruc et al., 2010), silica (Wen et al., 2017), gold NPs (Arvizo et al., 2012), black phosphorus (Qiu et al., 2019), and carbon-based nanomaterials (Panwar et al., 2019) have been developed for medical purposes. Robust and effective nanocarriers could even be responsive to multiplex combinations of diverse stimuli to further strengthen their specificity for targeted and controlled drug delivery (Biju, 2014).

**Figure 7:**
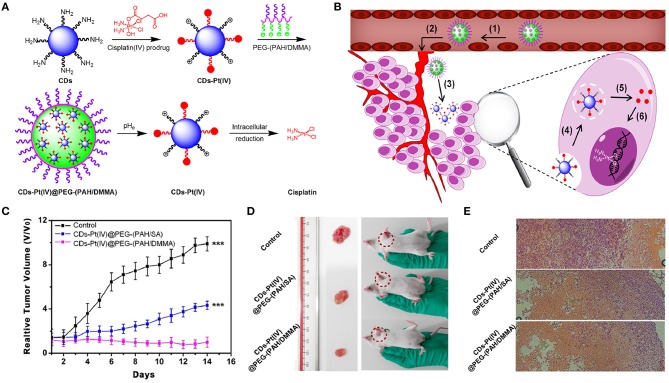
**(A)** Schematic illustration for the preparation of charge-convertible CD-based drug nanocarrier. pHe means tumor extracellular pH. **(B)** Schematic illustration for the drug delivery process: (1) negative charge/PEGylation to prolong circulation time, (2) accumulation at the tumor site through the EPR effect, (3) responsiveness to tumor extracellular pH, (4) effective uptake by cancer cells, (5) facilitated endosome escape by the “proton sponge” effect and controlled cisplatin release, and (6) cisplatin binding with DNA to exhibit cytotoxicity. **(C)** Relative tumor volume achieved from mice after intravenous treatments with CD-based drug nanocarrier. **(D)** Photographs of mice and excised tumors from representative euthanized mice. **(E)** H&E stained tumor slices from different groups after 14-day treatment. ^***^P < 0.001.

CDs can serve as imaging probes or as nanocarriers for transporting the targeted theranostic agents (e.g., PSs, drugs, genes) (Boakye-Yiadom et al., 2019). The surfaces of CDs, as the drug nanocarriers, are usually modified by negatively or positively charged PEG, which affects their therapeutic efficacy and future biological application. Owing to electrostatic repulsion from the same-charged cell membrane, negatively charged PEGylated CDs nanocarriers cannot easily enter the cancer cells, which affects intake and ultimately leads to low curative effects. Due to non-specific interactions with cellular ingredients (e.g., serum), positively charged CD carriers can be easily endocytosed through RES, resulting in quick clearance from blood circulation. Also, positively charged nanocarriers can be phagocytized by healthy cells to produce some potential toxicity via charge attraction with cell membrane (Mishra et al., 2018). To solve the above-mentioned problems, Feng et al. (2016) developed a cisplatin-loaded charge convertible CD nanocarrier functionalized by poly-(allyamine)-modified PEG, which could sensitively distinguish the pH difference between tumor pathologic outcomes and the normal physiological environment for controllable drug delivery (Figure 7A). The anionic poly-(allyamine)-modified PEG polymer with dimethylmaleic acid on the constructed CD nanocarrier would experience a charge transformation into the cationic polymer under the mild acidity of tumor cells (pH ~ 6.8), resulting in intense electrostatic repulsion and “leakage” of cisplatin(IV)-CD cations. Significantly, positive cisplatin(IV)-CDs showed high affinity for negative cancer cell membrane, thus leading to the enhancement of internalization and efficient release of drug in the reductive cytosol (Figure 7B). Tumors growth in mice injected with the drug-loaded CDs were greatly suppressed during 14 days of treatment (Figure 7C). The digital images of tumor excision in mice further confirmed that the sizes of the tumors were smallest using the drug-loaded CDs, indicating their better treatment efficiency (Figure 7D). Also, through hematoxylin and eosin (H&E) staining analysis of tumor tissues (Figure 7E), the drug-loaded CD treatment groups exhibited the greatest degree of cell injury, which was consistent with the tumor growth data.

Zinc gallogermanate structured PLNPs have demonstrated superior physiochemical properties, e.g., a strong NIR signal (quantum yield ~10%), superlong optical lifetime (>90 h), monodisperse stability, and minimal poisoning (Abdukayum et al., 2013). The superlong sustained emission and red light renewability of the trivalence-doped zinc gallogermanate PLNPs (ZGGO:Cr^3+^,Yb^3+^,Er^3+^) are the principal basis for long circulation bioimaging and drug release in living organisms (Li and Yan, 2016). Also, the easily controllable surface modulation of ZGGO PLNPs makes them desirable alternatives for fabricating the drug carriers. Liu et al. (2018b) incorporated red blood cell membrane vesicles with NIR PLNPs to construct an erythrocyte membrane bioexcited optical nanocarrier. In order to realize the biomimetic pattern, erythrocyte membrane vesicles were isolated from RBC and then fused with mesoporous SiO_2_-coated ZGGO to form the membrane bioexcited nanocarriers. Three groups of 4T1 tumor-bearing mice were individually treated with unmodified, SiO_2_-coated, or membrane-fused ZGGO via tail vein injection. The NPs were mainly distributed in the two primary RES organs (i.e., liver and spleen) and the tumors due to EPR effects. Meanwhile, compared with two other NPs, the membrane-fused ZGGO exhibited a far higher proportion of retention capacity. Moreover, the amounts of membrane-fused ZGGO loaded with the drug Dox in blood were measured, and the results indicated a long cycling period with a half-life of 9 h. Furthermore, injection of the drug-delivery vehicle into mice exhibited improved sustained-release efficacy of the NPs, strongly confirming the efficient decrease of systemic clearance and extension of circulation time caused by the biomimetic membrane coating. Employment of the drug-delivery system in the 4T1 orthotopic mammary tumor model showed the best tumor growth inhibition performance.

## Summary and Perspectives

By conjugating with different functional groups, ligands, and biomolecules, multifunctional NPs have displayed superb capabilities in diagnosis and therapeutic applications. Diverse designs and synthesis strategies have been developed to achieve specific NPs offering targeted and controlled drug delivery for practical use. Multiple aspects of NP research, from investigations of the effects of different kinds of NPs and their sizes on the imaging and therapeutic efficacy in biological environments to studies of specific ligand targeted drug delivery, have been carefully performed.

Despite these exciting achievements in the last few years, the distinctive functions of multifunctional nanomaterials have influenced the injection dose in different imaging models and therapy types. Therefore, the issues around the controllable synthesis of nanoagents, synergistic theranostic effects, and short-term and long-term toxicity should be carefully investigated as a whole to realize every function (diagnosis and therapy) and to achieve the lowest side effect with single injection.

More investigations are required to improve the performance of theranostic agents, such as intensifying excretion, strengthening continuous monitoring, prolonging blood circulation time, promoting physiological barrier penetration, evading the RES, and accelerating more materials into clinical trials. Therefore, continuous innovations and development for novel theranostic approaches are necessary to meet growing clinical requirements.

## Author Contributions

YY designed the whole structure of the article, wrote the sections on materials, bio-imaging, PDT, and reviewed the article. LW, BW, YG, and XL wrote the sections on PTT and drug delivery.

### Conflict of Interest

The authors declare that the research was conducted in the absence of any commercial or financial relationships that could be construed as a potential conflict of interest.
